# Enzyme-Like Properties of Gold Clusters for Biomedical Application

**DOI:** 10.3389/fchem.2020.00219

**Published:** 2020-04-03

**Authors:** Yunguang Zhang, Shuo Li, Haile Liu, Wei Long, Xiao-Dong Zhang

**Affiliations:** ^1^School of Science, Xi'an University of Posts and Telecommunications, Xi'an, China; ^2^Department of Physics and Tianjin Key Laboratory of Low Dimensional Materials Physics and Preparing Technology, School of Sciences, Tianjin University, Tianjin, China; ^3^Institute of Radiation Medicine, Chinese Academy of Medical Sciences and Peking Union Medical College, Tianjin, China

**Keywords:** gold nanoclusters, nanozyme, enzyme-like properties, cancer therapy, bio-detection

## Abstract

In recent years, the rapid development of nanoscience and technology has provided a new opportunity for the development and preparation of new inorganic enzymes. Nanozyme is a new generation of artificial mimetic enzyme, which like natural enzymes, can efficiently catalyze the substrate of enzyme under mild conditions, exhibiting catalytic efficiency, and enzymatic reaction kinetics similar to natural enzymes. However, nanozymes exist better stability than native enzymes, it can still maintain 85 % catalytic activity in strong acid and alkali (pH 2~10) or large temperature range (4~90°C). This provides conditions for designing complex catalytic systems. In this review, we discussed the enzymatic attributes and biomedical applications of gold nanoclusters, including peroxidase-like, catalase-like, detection of heavy metal ions, and therapy of brain and cancer etc. This review can help us understand the current research status nanozymes.

## Introduction

The natural enzyme is a kind of biocatalyst that is closely related to many life activities (Arnold et al., 2001). Similar to other catalysts, the presence of natural enzymes can greatly speed up the reaction and sometimes participate in the reaction, but the enzyme itself does not change before or after the reactions (Wilhelmová, 1996). In addition, it exhibits high catalytic efficiency, mild reaction conditions and high specificity (Sheldon, 2007). However, lots of natural enzymes are proteins, which are susceptible to high temperature, acidic or alkaline, causing loss of catalytic activity (Chang, 2013). In addition, natural enzymes presented in living organisms are difficult to purify, relatively expensive, and not easily transported and stored (Scopes, 2013). To overcome the limitations of instability and high cost for natural enzymes, researchers are looking for different ways to prepare mimetic enzymes to replace natural enzymes (Chen et al., 2010; Yang et al., 2016). Until now, different types of mimic enzymes have been designed and used in many biological fields, such as immunoassay (Lequin, 2005), glucose detection (Song et al., 2010), heavy metal ion detection (Kim et al., 2001), free radical protection (Barzegar and Moosavi-Movahedi, 2011), and tissue engineering (Griffith and Naughton, 2002). Host-guest chemistry (Wan et al., 2006) and supramolecular chemistry (Steed and Atwood, 2013) lay an important theoretical foundation for mimic enzyme. In essence, the basic meaning of host-guest chemistry comes from the interaction between enzyme and substrate, which is embodied in the complementary spatial and electronic arrangement of the binding site between the subject and the guest (Spichiger-Keller, 2008). This host-guest complementarity is similar to the combination of the nanozymes and the substrate. Based on non-covalent bond interactions, such as electrostatic interactions, hydrogen bonding and van der Waals forces (Pasternack et al., 1998; Wei and Wang, 2013), supramolecules are produced by the combination of substrate and receptor (Geim and Grigorieva, 2013). When receptors combine with complex ions or molecules, a supramolecule with stable structure and properties is formed, which has the functions of molecular recognition, catalytic and selective output. Host-guest chemistry and supramolecules chemistry are important theoretical weapons for the simulation of artificial enzymes.

At present, the ideal traditional enzyme systems include cyclodextrin (Del Valle, 2004), cyclophane (Gleiter and Hopf, 2006) and cyclic aromatic hydrocarbons (Tsipis and Tsipis, 2003). The chemical composition of traditional mimic *peroxidase-like activation* enzyme is non-protein, but these enzymes have similar catalytic performance to natural enzymes (Meeuwissen and Reek, 2010). These traditional mimetic enzymes are superior to natural enzymes in thermal stability and acid-base resistance, but their shortcomings, such as too-complex structure, difficulties in separation, single catalytic active sites, and low catalytic efficiency, still stunt their development progress (Liu Q. et al., 2017). With the development of nanomaterials, the researches on the catalysis of nanomaterials have also achieved many new results (Yin and Talapin, 2013). Nanozymes have the dual identities of enzymes and nanomaterials (Roduner, 2006). Unlike natural enzymes or traditional artificial enzymes, nanozymes (Wang et al., 2013; Li et al., 2015; Zhou et al., 2017; Fan et al., 2018; Qu et al., 2018; Chen et al., 2019; Huang et al., 2019a,b; Jiang et al., 2019; Liang and Yan, 2019; Liu et al., 2019; Mu et al., 2019a,b; Wu et al., 2019; Xu et al., 2019; Yan et al., 2019) have many physical and chemical properties, besides their catalytic functions (Senanayake et al., 2013). For example, Fe_3_O_4_ and CdS nanoparticles not only have the catalytic functions of peroxidase and superparamagnetic activities, but also have the property of luminescence (Liu et al., 2011). The enzymatic activity of nanozymes is not only related to its composition, crystal form and structure, but also to its surface properties (Lin et al., 2014a). Take gold nanoclusters (Au NCs) as an example, the combination of certain small molecules with Au NCs can change the surface microenvironment, resulting in changes in the catalytic activities of Au NCs (Jin, 2010). Wang et al. found that the surface modification of Au NCs can affect its catalytic activity (Yuwen et al., 2014). The Au NCs were modified with amino and citric acid respectively to make positive and negative charges on the surface, and then catalytically oxidize ABTS (Erel, 2004) (negatively charged) and TMB (Ding et al., 2018) (positively charged) to detect its catalytic activity. The amino and citric acid modified Au NCs were found to have high affinity to the substrates ABTS and TMB. Not only small molecules can be combined with Au NCs, inorganic nanoparticles, metal ions and biomacromolecules (such as DNA, RNA) can also be combined with it to change the surface microenvironment of Au NCs, thereby changing its catalytic activity. Compared with other nanomaterials with simulating peroxidase, gold nanoclusters have the advantages of small size, good stability, good biocompatibility, and are more prominent in the application of biological analysis. However, the potential of Au NCs as enzyme mimics is easily limited by the low catalytic activity at neutral environment. In addition, since the surface atom is the key catalytic sites for gold clusters, modification of nanozymes with various coating molecules may block their active sites, reducing or inhibiting their enzymatic activity. This review details the enzymatic properties of gold nanoclusters and their applications in biomedicine in recent years.

## Enzyme-Like Activity of Gold Clusters

### Peroxidase-Like Property of Gold Clusters

**Figure 1:**
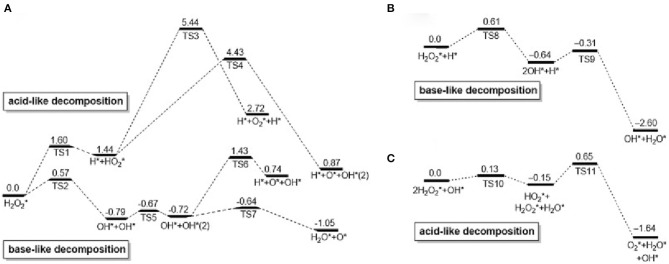
Calculated reaction spectrum (unit: eV) of H_2_O_2_ decomposition on Au surface: **(A)** neutral, **(B)** acidic, **(C)** basic [reproduced from Li et al. (2015) with permission from The Elsevier].

Peroxidase (Gao et al., 2007) is a kind of natural enzyme that have catalytic oxidation effects on hydrogen peroxide. The establishment of an analytical method involving H_2_O_2_ is of great significance in analytical chemistry and clinical medicine. In recent years, Au NCs have been reported to have peroxidase-like properties and are used in the fields of bionics, biosensing, and biomedicine (Feng et al., 2017; Liao et al., 2017). First, H_2_O_2_ can be adsorbed on the surface of gold nanomaterials, and the O–O bonds of H_2_O_2_ may be decomposed into dihydroxy radicals; at the same time, the generated hydroxyl radicals may be stabilized by gold nanomaterials through partial electron exchange interactions. This may contribute to their catalytic capabilities. Figure 1 shows the adsorption and decomposition of H_2_O_2_ on Au(111) under different pH conditions (Li et al., 2015). Under neutral conditions, H_2_O_2_ adsorbed on the surface of Au (111) can undergo acid decomposition and alkali decomposition. According to the principle of lowest energy, alkaline decomposition is more inclined under neutral conditions (Figure 1A) (He et al., 2012). It is worth noting that under these conditions, the adsorbed O^*^ cannot generate O_2_ under the high energy barrier of 1.42 eV (Wu et al., 2019). Under acidic conditions, the decomposition pathway of H_2_O_2_ is similar to the decomposition of alkalis under neutral conditions. First, OH^*^ is generated and then O^*^ and H_2_O^*^ are generated. The generated O^*^ can extract H from the substrate. Therefore, under acidic and neutral conditions, Au (111) has peroxidase activity (Figure 1B). Ding et al. used 3, 3′, 5,5′-tetramethylbenzidine (TMB) as a substrate and found that histidine-modified gold clusters (His-Au NCs) have peroxidase-like activity. When H_2_O_2_ is present, peroxidase can catalyze the oxidation of TMB. Reaction, when His-Au NCs is mixed with H_2_O_2_ and TMB, the solution rapidly changes from colorless to blue, and the maximum absorption wavelength of the mixture is 652 nm. This is due to the TMB is oxidized to oxTMB and the solution is blue. These results indicated that His-Au NCs are capable of oxidizing TMB to develop color and have peroxidase-like properties (Liu Y. et al., 2017). Lin et al. compared the catalytic activity of unmodified Au NCs and studied the effect of amino-modified gold clusters (NH_2_-Au NCs) and citric acid-modified gold clusters (itrate-Au NCs) on the substrates ABTS and TMB. It was found that the surface unmodified Au NCs has the highest peroxidase activity. NH_2_-Au NCs have higher catalytic activities for ABTS than citrate-Au NCs, while NH_2_-Au NCs have lower catalytic activity for TMB than itrate-Au NCs. For the surface modified by Au NCs has different charges, it exhibits different variation, and the ability to adsorb the oxidized substrate is different, thus showing the difference in catalytic activity. The biocompatibility of Au NCs was adjusted by selecting different ligands (Liu et al., 2016). *Wang's team* prepared bovine serum albumin (BSA) modified Au NCs. In order to improve the peroxidase- like activity of Au NCs (Wang et al., 2011). Wen et al. used the horseradish peroxidase properties of gold nanoclusters to detect H_2_O_2_ (Wen et al., 2011). Jiang et al. discovered and applied precious metal nanoclusters. They report that gold chains of ferritin iron (Au-Ft) can produce a blue reaction by catalyzing the oxidation of TMB by H_2_O_2_. Compared to the native enzyme, Au-Ft exhibits higher activity and pH, temperature range, and the catalyzed reaction follows typical Mie kinetics. The lower K_m_ value (0.097 μM) was exhibited by the Au-Ft kinetic parameters, and the specific activity for TMB oxidation exceeded HRP. According to these findings, Au-Ft was used as a peroxide mimic enzyme to perform glucose spectrometry. Photometric analysis the system exhibited acceptable repeatability and high specificity (Liu J. et al., 2017).

### Catalase-Like Property of Gold Clusters

**Figure 2:**
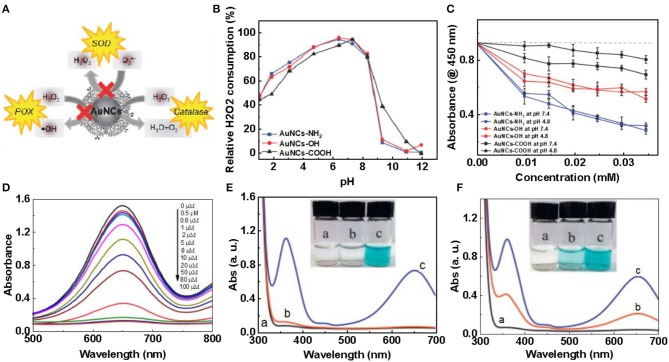
Catalase-like of gold clusters **(A)** Au NCs-NH_2_ can catalyze H_2_O_2_ to produce H_2_O and O_2_ through catalase-like activity. **(B)** Consumption of H_2_O_2_ by Au NCs-NH_2_, Au NCs-OH and Au NCs-COOH at the same concentration (0.02 × 10^−3^ M). **(C)** The catalytic activities of different concentrations of Au NCs-NH_2_, Au NCs-OH and Au NCs-COOH on H_2_O_2_ (0.1 × 10^−3^ M) at pH 4.8 and 7.4 [reproduced from Liu C. P. et al. (2017) with permission from The Small]. **(D)** Effects of different concentrations of Ag^+^ on catalase activity of BSA-Au NCs. Red BSA-Au NCs [reproduced from Chang et al. (2016) with permission from The Elsevier]. **(E)** And blue BSA-Au NCs. **(F)** Catalyze TMB under different conditions: (a) under visible light, (b) in the presence of Au NCs, (c) in the presence of Au NCs under visible light [reproduced from Wang et al. (2015) with permission from The Elsevier].

Catalase is an important enzyme that prevents oxidative damage of cells by reactive oxygen species (Glorieux and Calderon, 2017). For Au NCs, Au^2+^ is first reduced by a H_2_O_2_ to form Au^+^, which is accompanied by the production of protons and O_2_. After that, another H_2_O_2_ can be combined with oxygen vacancies to oxidize Au^+^ to Au^2+^ and release H_2_O. This completes the simulation of hydrogen peroxide. Most nanomaterial-based peroxidase mimetics typically exhibit enzymatic activity under alkaline conditions rather than under physiological conditions (Góth et al., 2004; Glorieux and Calderon, 2017). Under the basic conditions of OH pre-adsorption, H_2_O_2_ first transfers one H^*^ to the pre-adsorbed OH to form HO${}_{2}^{*}$ and H_2_O^*^; then, HO${}_{2}^{*}$ gives one H to the other H_2_O_2_ and finally produces H_2_O ^*^ and O_2_^*^ (Figure 1C). Therefore, hydrogen peroxide-based activity can be observed under alkaline conditions (Li et al., 2015). He et al. (2013) demonstrated the intrinsic catalase activity of Au NCs using electron spin resonance spectroscopy combined with spin trapping and spin labeling. Under normal and basic conditions, Au NCs exhibit inherent catalase catalytic activity because Au NCs can convert H_2_O_2_ to H_2_O and O_2_. However, under acidic conditions, the catalase-like activity of Au NCs is significantly reduced, and once trapped in organelles, such as endosomes (pH≈5.5) and lysosomes (pH≈4.8), hydroxyl groups are produced. Free radicals (**·**OH) can easily induce apoptosis. Therefore, the catalase-like activity of Au NCs is limited (He et al., 2013). To change this situation, Liu et al. studied amine-terminated macromolecularly encapsulated gold nanoclusters (Au NCs-NH_2_). Au NCs-NH_2_ exhibits good catalase activity at physiologically acidic pH values (Liu C. P. et al., 2017). Figure 2A is a schematic diagram showing the enzymatic activity of Au NCs-NH_2_, which can catalyze the production of O_2_ by H_2_O_2_ by catalase activity. They changed the different groups of dendrimers, added Au NCs-OH and Au NCs-COOH, and studied the relative H_2_O_2_ consumption of Au NCs-NH_2_, Au NCs-OH and Au NCs-COOH under different solution pH values. A comparison of the amounts, they still have significant catalase-like activity at pH 4.8-7.4 (Figure 2B). By comparing the catalytic activities of Au NCs-NH_2_, Au NCs-OH and Au NCs-COOH at various concentrations of H_2_O_2_ at pH 4.8 and pH 7.4, the effect of Au NCs-NH_2_ was significantly better (Figure 2C). Fan et al. synthesized a derivative protein (apoFt) as a nanoreactor to obtain Au-apoFt with adjustable size and uniform dispersion. The catalytic activity of both was observed at pH and temperature compared to natural catalase. As the pH and temperature increase, the enzyme activity of Au-apoFt increases. The increase in pH may be due to the presence of OH- which promotes the dehydrogenation step of water in the catalytic reaction, showing an increase in enzyme activity. With rising of temperature, the increase in the enzyme activity of Au-apoFt may be due to an increase in the rate of molecular motion, which allows more H_2_O_2_ to adsorb to the surface of the Au nanoclusters, showing an increase in catalytic activity. Liu et al. found that bovine serum albumin-protected gold nanoclusters (BSA-Au NCs) can sensitively measure H_2_O_2_ concentration (Figure 2D). Red BSA-Au NCs have no catalytic activity for TMB in the presence of oxygen but no light, and have catalytic activity in the presence of oxygen and light (Figure 2E); blue BSA-Au NCs have a slight catalytic activity for TMB in the presence of oxygen but no light, and have strong catalytic activity when oxygen and light coexist (Figure 2F). The experiment shows that light can stimulate the catalytic activity of Au NCs (Wang et al., 2015).

### Glucose Oxidase-Like Property and Superoxide Dismutase-Like Property of Gold Clusters

**Figure 3:**
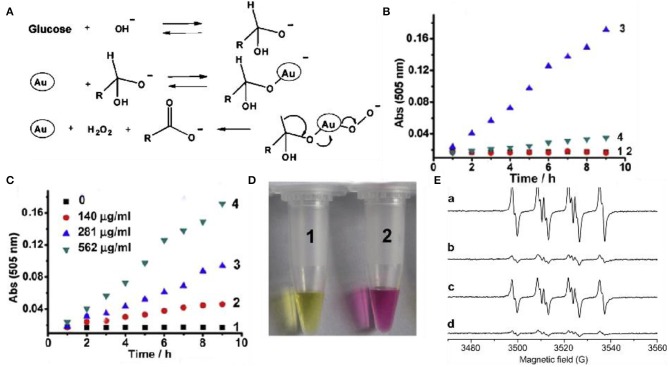
Glucose oxidase-like and superoxide dismutase-like of gold clusters **(A)** Catalytic mechanism of GOD-like enzymes of Au nanoparticles [reproduced from Lin et al. (2014a) with permission from The Wiley-VCH]. **(B)** The oxidative decomposition of glucose into gluconic acid, absorbance at 505 nm. (1) Glucose only (2) EMSN-Au NPs alone (3) Glucose and EMSN-Au NPs (4) Glucose and EMSN-Au NPs. **(C)** In the presence of different concentrations of EMSN-Au NPs, glucose is oxidative decomposed into gluconic acid, the absorbance at 505 nm. **(D)** Typical photo of methyl red solution of glucose and EMSN-Au NPs [reproduced from Lin et al. (2014a) with permission from The Elsevier]. **(E)** Au NPs scavenge SOD-like activity of superoxide [reproduced from He et al. (2013) with permission from The Elsevier].

Glucose oxidase (GOD) is widely distributed in animals and plants and microorganisms, and can specifically catalyze the production of glucose into gluconic acid and hydrogen peroxide under aerobic conditions (Gibson et al., 1964; Wilson and Turner, 1992; Bankar et al., 2009; Luo et al., 2010). At present, the application fields of GOD are constantly expanding, and the demand in domestic and foreign markets has increased dramatically. Low yield, low enzyme activity, and complex detection methods are the limiting factors for GOD industrialization. A lot of work has been done at home and abroad and significant progress has been made. Recent studies have proved that gold clusters have excellent GOD-like enzyme activity. Luo et al. reported an interesting autocatalytic, self-limiting system that controls the controlled growth of Au NCs with GOD-like (Luo et al., 2010). In this system, Au NCs can serve as both seeds and catalyst, that is, the Au NCs catalyzed glucose oxidation *in situ* produced H_2_O_2_, and induced the Au NCs seeds in the presence of gold chloride ions. More importantly, the growth of Au NCs is internally regulated by two negative feedback factors, the reduced size-dependent activity of Au NCs and the glucose-induced surface passivation of the products, leading to rapid self-limiting systems. Pandey et al. (2007) used chemical synthesis to covalently combine GOD with the surface of gold nanoclusters to form a GOD-Au NCs complex, which improves the catalytic activity of GOD, improves stability, and enhances the enzyme response temperature and pH durability (Xia et al., 2013). However, this method only improves the performance of the enzyme and does not fundamentally solve the problem. The activity of the enzyme is still restricted by a series of factors, and other substances are introduced into the reaction system, which makes the entire system more complicated. Rossi et al. found that in the presence of O_2_, glucose can be catalyzed by “bare” gold nanoclusters to produce gluconic acid and H_2_O_2_ (Pina et al., 2011). Based on the promotion of alkali and the production of H_2_O_2_, they proposed the mechanism of molecular activated gold catalysis (Figure 3A). The surface of the gold atom interacts with the hydrated glucose anion to form electron-rich gold, which effectively activates molecular oxygen through nucleophilic attack. O_2_ and gold intermediates Au^2+^ + -O${}_{2}^{2-}$ or Au + -O${}_{2}^{-}$ can act as a bridge for the conversion of electrons from glucose to hydrogen peroxide. Thereby the final reaction product is formed. Lin et al. studied mesoporous silica-encapsulated gold nanoclusters (EMSN-Au NPs) with GOD properties through UV-Vis (Figures 3B,C) (Lin et al., 2014a). First, the GOD mimicking activity of EMSN-Au NPs in solution was evaluated. Glucose is catalyzed by them in the presence of O_2_ to produce gluconic acid. During the experiment, they used methyl red to detect the change in pH of the solution, as shown in Figure 3D. The results further confirmed that gluconic acid was indeed produced in the reaction catalyzed by EMSN-Au NPs. Superoxide dismutase (SOD) is a kind of antioxidant metal enzyme *in vivo*. It can catalyze superoxide anion free radical disproportionation to generate hydrogen peroxide and oxygen, which is very important in anti-oxidation. Gold nanoclusters decompose O^2−^ into molecular oxygen (O_2_) and hydrogen peroxide (H_2_O_2_) through a cyclic redox electron transfer mechanism, thereby eliminating O${}_{2}^{-}$ activity. Weiwei et al. verified the SOD activity of Au NPs through ESR experiments (Figure 3E) (He et al., 2013).

### Effect of Support and Particle Size on Gold Cluster Activity

**Figure 4:**
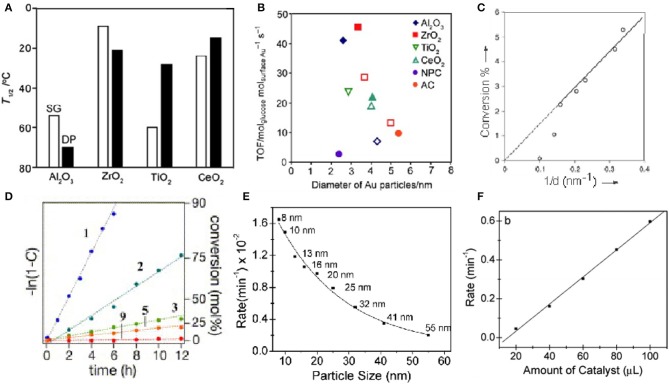
Effect of support and particle size on gold cluster activity **(A)** Catalytic performance of different supported gold nanoclusters for CO. **(B)** Turnover frequency of glucose oxidation [reproduced from Ishida et al., 2008 with permission from The Wiley-VCH]. **(C)** Relationship between conversion rate and particle size [reproduced from Comotti et al. (2010) with permission from The Wiley-VCH]. **(D)** Relationship between the catalytic activities of Au: PVP clusters for the aerobic oxidation of hydroxybenzyl alcohol and the nuclear size [reproduced from Tsunoyama et al. (2006) with permission from The Elsevier]. **(E)** Graph of reduction rate as a function of R-Au particle size. **(F)** Relationship between different volumes and reaction rates [reproduced from Panigrahi et al. (2007) with permission from The American Chemical Society].

The enzymatic activities of nanomaterials are related to size (Mavrikakis et al., 2000; Lopez et al., 2004; Molina and Hammer, 2005; Miller et al., 2006; Zhou et al., 2010; Brodersen et al., 2011). It is possible to regulate the activity of nanomaterials by controlling their size, which has been confirmed in many studies. For gold nanomaterials as an example; the enzyme-like activity of gold nanoclusters is stronger than that of gold nanoparticles. Li et al. compared the enzyme-like activities of gold nanozymes with different structures and morphologies, including Au nanoclusters, Au nanoparticles, and Au nanotubes. At the same specific surface area, the Au nanoclusters had the strongest enzyme-like activity, while the Au nanotubes had the weakest enzyme-like activity. Through the study and analysis, they concluded that the differences in the enzyme-like activity of materials may be related to the differences in the crystal planes (Li et al., 2015). Corma et al. supported gold atoms on functionalized carbon nanotubes and explored their catalytic properties for phenol. They have catalytic activity equivalent to that of thiol oxidase. The catalytic activity also decreases with the increase of the size of the gold cluster, until it almost disappeared. According to theoretical calculations, smaller gold clusters can activate thiophenol and O_2_ and are therefore active, while larger nanoparticles are inactivated by alkoxides and lose their activity (Corma et al., 2013). Tamao et al. tested the catalytic performance of gold nanoparticles on CO under different loads (Figure 4A). Compared with ZrO_2_, TiO_2_ and CeO_2_, Al_2_O_3_ has a lower catalytic activity (Ishida et al., 2008). Oxygen vacancies can be formed at the peripheral interface of the Au particles. When gold nanoclusters are deposited on carbon materials and polymers, they lose their catalytic activity at temperatures above 120°C In the CO oxidation reaction, the catalytic activity of glucose oxidation observed on Au catalysts is more affected by Au particle size than by carrier properties, and is related to the turnover frequency (TOF) of Au atoms on the surface (Figure 4B). Rossi et al. studied the effect of particle size on the catalytic activity by using gold clusters (3–10nm) dispersed in water (Comotti et al., 2010). According to their research, the catalytic activity of glucose oxidase increases with decreasing particle size (Figure 4C). They first showed that gold nanoclusters (Au NCs) can catalyze the oxidation of glucose in the presence of O_2_ to produce gluconic acid and H_2_O_2_. In contrast, other metal nanomaterials tested, such as Cu, Ag, Pd, and Pt, did not show significant oxidase-like activity under similar conditions (Comotti et al., 2010; Quan et al., 2015). Lin et al. compared the CAT activity of several clusters experimentally, and the results showed that the CAT activity of Pt and Pd was better than that of Au and Ag. Otherwise, the SOD-like and CAT-like activity of Au and Pt nanozymes increased under alkaline conditions and decreased under acidic conditions (Lin et al., 2014b). Tsunoyama et al. prepared a group of monodisperse gold clusters (Au: PVP) through seed-mediated growth in the presence of polyvinylpyrrolidone (PVP) (Tsunoyama et al., 2006). The catalytic activity of Au: PVP clusters on hydroxybenzyl alcohol decreases with increasing core size (Figure 4D). Panigrahi et al. studied a core-shell Nano composite (R-Au) (Figure 4E) and derived the relationship between reduction rate and gold nanoclusters size (Panigrahi et al., 2007). The reaction rate decreases with increasing particle size. When the particle size is increased to 32 nm, as the particle size increases, the decline rate tends to be gentle. They believe that as the particle size increases, the decrease in catalytic performance is due to the increase in particle surface roughness. Therefore, the smaller the particle size, the higher the catalytic activity (Figure 4F).

## Application of Gold Clusters

### Detection of Heavy Metal Ions

**Figure 5:**
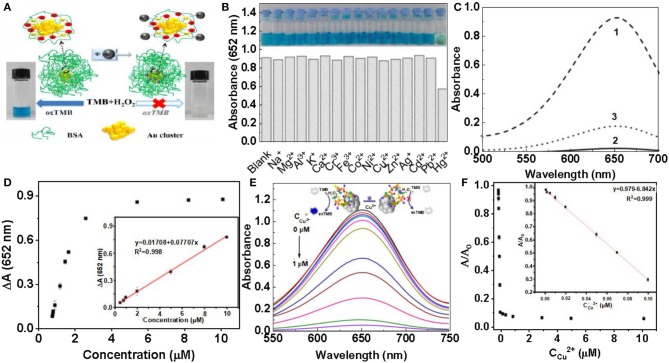
Detection of heavy metal ions **(A)** Schematic diagram of Hg^2+^ detection principle. **(B)** The effect of different metal ions on the peroxidase like activity of BSA-Au NCs. **(C)** The inhibition of Hg^2+^ on the activity of BSA-Au NCS peroxidase under different conditions [reproduced from Zhu et al. (2013) with permission from The Elsevier]. **(D)** Hg^2+^ can specifically interact with Au^+^ and inhibit enzyme activity [reproduced from Chang et al. (2016) with permission from The Elsevier]. **(E)** Effect of different concentrations of copper ions on the absorption strength of solution. **(F)** A/A_0_ plot plotted at the concentration of Cu^2+^. A and A_0_ are absorbance at 652 nm with and without copper ions [reproduced from Liu Y. et al. (2017) with permission from The Elsevier].

At present, heavy-metal contamination has caused great threats to the human health and our living environment. It is of great significance for the detection of heavy metals, with high selectivity of heavy metals (Bhan and Sarkar, 2005; Gallardo et al., 2014; Martin and Griswold, 2018). It is well-known that mercury is a toxic heavy metal and widely found in the environment (Ercal et al., 2001). Mercury ion (Hg^2+^) is the most common heavy metal ions. Even at very low concentrations, its destructive properties can affect the brain, god system and kidney (Bhan and Sarkar, 2005). Therefore, it is necessary to establish a fast, simple and sensitive method to detect Hg^2+^ in the environment. Zhu et al. found that Hg^2+^ has a selective inhibitory effect on the peroxidase activity of BSA-Au clusters (Zhu et al., 2013). The effect of common metal ions on the catalytic activity of BSA-Au was investigated using fluorescence quenching (Figure 5A). At the same concentration, Na^+^, Fe^3+^, Co^2+^, Ag^+^, Mg^2+^, Al^3+^, K^+^, Ca^2+^, Cr^3+^, Ni^2+^, Cu^2+^, Zn^2+^, Cd^2+^, Pb^2+^ to BSA-Au NCs have no effect on peroxidase-like activity. Hg^2+^ can inhibit the peroxidase-like activity of BSA-Au NCs and hardly catalyze the color reaction of TMB and H_2_O_2_ (Figure 5B). Similarly, the absorption spectrum of the reaction solution at 652 nm can also indicate that Hg^2+^ can inhibit the catalytic activity of BSA-Au NCs (Figure 5C, curve 2). Chelation of EDTA with Hg^2+^ can reduce the inhibitory effect of Hg^2+^ on the catalytic activity of BSA-Au NCs (Figure 5C, curve 3). Because mercury is easily complexed with sulfur, mercury ions (Hg^2+^) can combine with cysteine through Hg-S bond to form Hg-Cys complex. The affinity of cysteine to Hg^2+^ was significantly higher than that of other metal ions. Based on this mechanism, Ding et al. constructed fluorescence quenching of citrate-modified Au NCs to detect Hg^2+^ in tap water (Ding et al., 2012). Then, Qi et al. reported a probe that can paper-based visualization of Hg^2+^ based on conjugates of Tb^3+^/BSA-Au NCs. The probe can be highly complexed with Hg^2+^ via Hg-S bonds, and therefore has excellent selectivity. The method is simple and easy to operate, with only a ultraviolet lamp needed, which can be greatly promoted in practical applications (Qi et al., 2015). Using similar sensing mechanism, Lin et al. constructed a method to detect Hg^2+^ and methylmercury in seawater based on Lys protected Au NCs probe, and Xu et al. also used lysozyme-modified Au NCs to visually detect Hg^2+^ in water by visual and fluorescent colorimetry (Xu et al., 2015). The Zhu working group provided us with a colorimetric method that can detect Hg with high sensitivity and selectivity. They also detected of different working fluids and the influence of common metal ions on the catalytic activity. Finally, Hg^2+^ can specifically interact with Au^+^ to inhibit enzyme activity (Figure 5D) (Zhu et al., 2013). Liu et al. combined the peroxidase-like nanozymes activity of gold nanoclusters with the double-stranded nature of amino acids, and proposed a simple, sensitive and selective method for the detection of Cu^2+^ and histidine (His). The addition of different concentrations of Cu^2+^ can inhibit the peroxidation of histidine-gold nanoclusters (His-Au NCs) to varying degrees (Figure 5E). The absorbance of the solution at 652 nm gradually decreases with increasing Cu^2+^ concentration, so this method can be used to detect the concentration of Cu^2+^ in the solution. According to this calibration curve, Cu^2+^ can be measured with high sensitivity (Figure 5F) (Liu Y. et al., 2017). Although gold nanocluster probes with enzyme activity have been widely developed in recent years, many problems still need to be solved. First, most of the probes with enzyme activity controlled by metal ions are POD activity, while few probes are available for other types of enzyme activities. In addition, the sensor mechanism of the probes is relatively single, and more types of mechanisms need to be developed to design the probes. Finally, there is little research on the application in biological matrix or *in vivo*, which is very important for the biological application of enzyme activity.

### Anion Detection

Inorganic anions are widely present in ecosystems, but it should be noted that most inorganic anions have two-sided effects on the ecological environment and human health (Velizarov et al., 2004). Shojaeifard et al. tested environmental water and CN in human serum based on a combination of Au NCs and copper (II)-phthalocyanine complexes. Under the condition of CN^−^, the binding of Au NCs to the copper (II)-phthalocyanine complex is destroyed, so the fluorescence of the copper (II)- phthalocyanine complex quenched by Au NCs is restored, thus forming a stable [Au (CN)_2_]^−^. The method has good selectivity, high sensitivity and is suitable for popularization (Shojaeifard et al., 2016). Liu et al. also constructed a method based on Au NCs to detect CN^−^ in environmental water samples, and successfully used to detect CN^−^ in food sand samples and biological samples (Liu et al., 2010). Xiong et al. synthesized BSA-Au NCs and successfully detected Cl^−^ in tap water with a detection limit of 0.50 μmol/L (Xiong et al., 2015). Wang et al. used glutathione-coated Au NCs as probes to construct a method for detecting I^−^ in water with a detection limit of 400 nmol/L. This method can selectively identify I^−^ from 12 common anions such as F^−^, Cl^−^, Br^−^ (Yang et al., 2014). At the same time, Chang group used DNA as template to synthesize gold/silver nanozymes. Clusters were used to detect S^2−^ in hot spring and seawater samples. This method specifically recognized S^2−^ from Au^3+^, Ag^+^ and DNA in the presence of NaBH_4_. The quenching mechanism is that S can interact with gold and silver atoms, thus resulting in changes in the conformation of the template DNA (Chen et al., 2011). At present, gold clusters have been used to detect anions *in vitro* more thoroughly and sensitively, but few studies have been done in combination with organisms. The biological environment is more complex, so anion detection *in vivo* will be a challenge. In addition, the role of gold clusters in organisms should not only be a function of detection, but also require us to develop more properties of gold clusters for the diagnosis and treatment of organisms.

### Biological Application of Gold Clusters

#### Tumor Treatment

**Figure 6:**
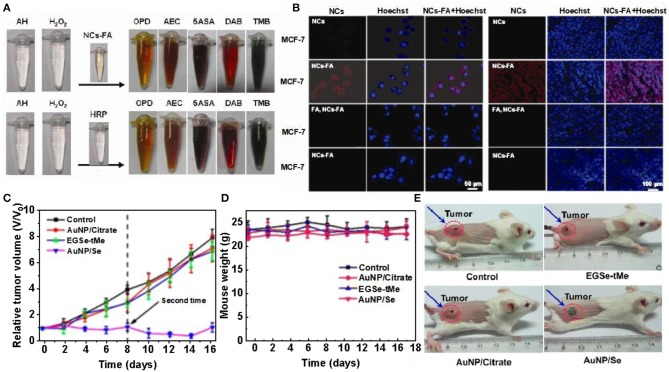
Gold clusters for tumor treatment. **(A)** NCs-FA and HRP catalyze the color contrast of OPD, AEC, 5ASA, DAB, and TMB. **(B)** MCF-7 cells and HepG2 cells were stained with NCs-FA under a confocal laser microscope [reproduced from Hu et al. (2014) with permission from The Ivy Publisher]. **(C)** Fluorescence imaging of NCs-FA stained MCF-7 and HepG2 tumor tissues. **(D)** Relative tumor volume of mice after different drug treatments. **(E)** Body weight changes in mice after different drug treatments [reproduced from Li et al. (2016) with permission from The American Chemical Society].

Nanoprobe with enzymatic properties have been attracting increasing attention in early screening and diagnosis of cancer (Schaller and Graf, 2004; Spichiger-Keller, 2008; He et al., 2010; Chi et al., 2012; Li and Xiaogang, 2015). In order to achieve the specificity and high accuracy of tumor detection, it was necessary to design and prepare an enzyme-simulated nanoprobe with tumor targeting, high enzyme activity and containing luminescent properties. Hu et al. described the folate receptor based gold nanoclusters (NCs-FA), which are novel luciferase mimic nanoprobes with high stability, low cytotoxicity and high enzyme activity. O-Phenylenediamine (OPD), 3-amino-9-ethylcarbazole (AEC), 5-aminosalicylic acid (5ASA) and 3,3,5,5-tetramethylbenzidine (TMB) can be used NCs-FA catalyzes to produce red, brown, brown, and blue. Experiments prove that NCs-FA nanoprobe has peroxidase activity (Figure 6A). To visualize the uptake of NCS-FA nanoprobes by cells, MCF-7 and HepG2 cells were stained with NCs-FA nanoprobes (NC) and observed under a focused laser microscope (Figure 6B) (Hu et al., 2014). The probes (NC) stained MCF-7 and HepG2 tumor tissues and observed them by fluorescence microscopy (Figure 6C). It confirmed that NCs-FA nanoprobes target tumor cells *via* FR. Au NPs have low anticancer activity and are widely used in drug carriers, biological imaging, and other fields. The new selenium-containing molecule (EGSE-TME) has low anticancer activity, but the combination of NCs-FA and EGSE-TME has produced a system with good anticancer activity. Li et al. synthesized Au NP/Se. In order to explore its cytotoxicity, tumor mice were injected with PBS, Au NP/citrate, EGSe-tMe, and Au NP/Se on day 0 and 8, respectively. Compared with the tumor volume of the control group, mice treated with Au NP/ citrate, EGSe-tMe had less obvious tumor growth inhibition effect, and however, mice treated with Au NP/Se showed tumor growth strong inhibitory effect (Figure 6D). There was no significant cha of mice treated with Au NP/Se (Figure 6E). And photographs of mice after administration of different drugs on day 10 were observed, which showed that the systemic toxicity of Au NP/Se was low (Li et al., 2016). However, there are still some problems in the development of nanoprobe for tumor microenvironment. Further binding to ligands may help to reduce toxicity and guide targeting, but can affect the catalytic activity and subsequent metabolism of the nanozymes, as well as the microenvironment of the organisms. Although nanoprobes have achieved good results in animal experiments, the differences between animals and humans are huge, and their clinical applications need to be further developed and utilized.

#### Brain Therapy

**Figure 7:**
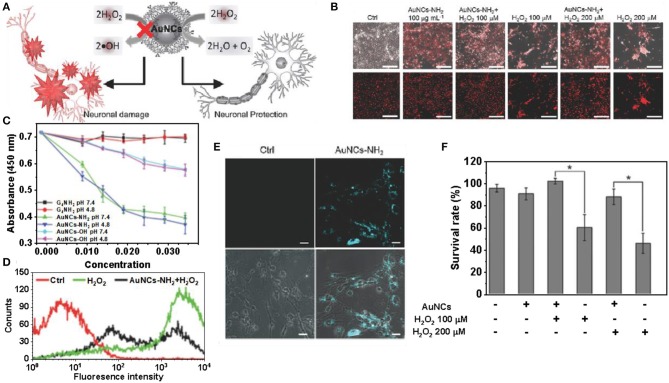
Gold clusters for brain disease treatment. **(A)** Au NCs coated with dendrimers have the effect of detoxifying H_2_O_2_ to protect neurons from oxidative damage. **(B)** The H_2_O_2_ challenge resulted in significant cell death, with less red fluorescence than the control. **(C)** Cell viability of neurons pretreated with AuNCs-NH_2_ under H_2_O_2_. **(D)** The catalytic activities of G_4_NH_2_, Au NCs-OH and Au NCs-NH_2_ on H_2_O_2_ were compared at pH 4.8 and 7.4, respectively. **(E)** Changes in DCF fluorescence intensity in neurons pretreated with Au NCs-NH_2_. **(F)** Confocal fluorescence images of neuronal cells treated with Au NCs-NH_2_ for 24 h [reproduced from Liu et al. (2016) with permission from The Small].

The toxicity of nanozymes has caused widespread concern (Zhang et al., 2018). Liu et al. found that amino-terminated gold nanoclusters (Au NCs-NH_2_) have low cytotoxicity and can protect primary neurons from oxidative damage (Liu et al., 2016). The peroxidase-like activity of Au NCs-NH_2_ is inhibited by the polymerized 3-amines, and thus exhibits catalase-like activity, which decomposes H_2_O_2_, thereby providing neurons with protection against oxidative damage (Figure 7A) (Mukherjee et al., 2010). Compared to the control, H_2_O_2_ excitation resulted in significant cell death and less red fluorescence. Neuronal cells were pre-treated with Au NCs-NH_2_ and showed red fluorescence similar to that of the control group after challenge with H_2_O_2_. The same is true for neurons treated with Au NCs-NH_2_ alone, further demonstrating the low cytotoxicity of Au NCs-NH_2_ (Figure 7B) (Wang et al., 2013). Cell viability in different treatment groups was determined by MTT assay and treatment with H_2_O_2_ alone (100 × 10^−6^ or 200 × 10^−6^ M) resulted in a significant decrease in neuronal cell viability. Pretreatment with Au NCs-NH_2_ followed by challenge with H_2_O_2_, the cells remained viable and the results were similar to the untreated controls. This indicates that pretreatment of primary neuronal cells with Au NCs-NH_2_ have resistance to H_2_O_2_ induced toxicity (Figure 7C) (Huang et al., 2004). The catalase-like activities of Au NCs-NH_2_ and Au NCs-OH prepared from different end groups of dendrimers were not affected at different pH, but the catalytic performance of Au NCs-NH_2_ for H_2_O_2_ was significantly better than that of Au NCs-OH (Figure 7D) (Jao et al., 2010). The level of H_2_O_2_ in cells after H2DCFDA staining was quantified by flow cytometry, and the protective effect of NCs-NH_2_ in H_2_O_2_ treated neurons was obtained (Figures 7E,F). The fluorescence intensity of neurons pretreated with Au NCs-NH_2_ was significantly increased compared to the control (Graf et al., 2004). Although the catalytic activity of nanozymes is closely related to their surface properties, the mechanism of enzymelike activity of its surface coating is poorly understood. Once the nano-enzyme loses its surface accessibility, its catalytic performance will be inhibited, thus affecting its application in brain therapy and other aspects. Currently, biomedical research on gold nanoclusters without surface coating is limited due to concerns about their toxicity and stability.

## Conclusion

In this article, we attempt to give a comprehensive overview based on gold nanoclusters. We have summarized the characteristics of enzymes such as glucose oxidase, peroxidase, catalase, superoxide dismutase, etc. The applications of gold nanoclusters in ion detection, tumor treatment and brain treatment were analyzed. This enzymatic property of gold clusters is derived from the functional groups present on the gold itself or on the surrounding monolayer. Although the development of gold clusters catalysts has made encouraging progress, the overall performance of these artificial catalytic systems is often not comparable to natural catalysts. To this end, the following aspects are awaiting implementation:

1. In recent years, with the development of nanotechnology, we can control the size and shape of nanoparticles by including hydrogen reduction, self-assembly, porous support matrix and surfactant assisted methods to adjust their catalytic activity. Because of the size dependence of gold nanomaterials, this allows us to conduct directional manipulation of surface properties. Because the surface charge and other parameters have great influence on the cell adhesion behavior, the preparation of gold nanoclusters enzyme with atomic precision is a promising method to affect the biological activity. In addition, catalysts with atomic precision are more conducive to revealing the relationship between electronic structure and properties. At the same time, the sources of catalytic activity of functional groups, and the future work may continue to focus on changes in functional groups present at catalytic sites, resulting in highly active catalysts. More importantly, we hope to open up new strategies to significantly improve catalytic performance and biosafety through the regulation of three changes in atomic accuracy, catalytic sites and functional groups.
2. Although rapid progress has been made in the development of nanomaterials with enzyme mimicking activity, it is unclear how widely these materials are used *in vivo* and clinically. So far, it is uncertain whether gold nanoclusters with enzyme-like activity can significantly replace many naturally occurring enzymes. All endogenous enzymes in our bodies are as a whole system and work interdependently. Any artificial substitutes, including nanoclusters, may not be suitable for the system and may cause serious side effects. During the expression of enzyme-like functions, nanoclusters can generate free radicals, causing toxicological effects. Therefore, it is worthy of further study to make full use of the beneficial effect of the enzyme-like activity of the nanoclusters and explore their application *in vivo*.
3. Although many researchers have demonstrated the therapeutic effects of nanozymes systems through cell and animal experiments, it is still difficult to explain the mechanism of nanozymes systems *in vivo* through these biological experiments. For example, quantifying reaction kinetics *in vivo* is not an easy task, although the available evidence supports the ultimate therapeutic outcome of these nanozymes. We can only know the results, but not the processes in the body. In recent years, multifunctional optical probes have been widely developed to detect the concentration and distribution of specific biochemical substances in the biological environment. Therefore, we may need to develop multifunctional nanometer enzyme probes, such as using the luminescence of gold clusters for imaging or sensing (Such as detecting markers of neurological disease), and then combining its enzyme-like properties for treatment, which can achieve the purpose of combining diagnosis with treatment, providing powerful help for the study of the distribution and mechanism of action of nanomaterials in living organisms. These diagnostic reagents are expected to help accurately characterize the catalytic process *in vivo*, and ultimately comprehensively clarify the relationship between the composition, structure, and *in vivo* properties of nanozymes drugs.

## Author Contributions

SL wrote the first draft. HL and X-DZ modified the manuscript content and format. YZ and WL modified the syntax.

### Conflict of Interest

The authors declare that the research was conducted in the absence of any commercial or financial relationships that could be construed as a potential conflict of interest.
